# “I Can’t Cope” the Use of ECT for Treatment Resistant Depression in a Patient With Other Psychiatric comorbidities

**DOI:** 10.1192/bjo.2026.11896

**Published:** 2026-06-30

**Authors:** Sabina Smith, Mani Krishnan

**Affiliations:** Tees, Esk and Wear Valleys NHS foundation trust, Stockton, United Kingdom

## Abstract

**Aims:**

Regardless of comorbid personality disorder there is value in use of ECT for treatment resistant depression. We present a case of a patient with treatment resistant depression and dependent personality disorder.

There is evidence that ECT used to treat depression with a comorbid personality disorder has poorer outcomes. Ferrea et al., 2023, found poor response rates in comorbid personality disorder as opposed to depression alone, with increased risk of relapse, but still showed a remission rate of 1 in 3 and 50% response rate. However, most of the studies included didn’t differentiate the type of personality disorder, leaving us without clear evidence around the impact of dependent personality disorder specifically.

**Methods:**

We present a case of a 78-year-old female patient with diagnoses of recurrent depressive disorder and generalised anxiety disorder. The patient had two admissions in two years under the mental health act with similar presentations in keeping with severe depression. In the community she was being investigated for autism, underlying cognitive impairment, dependent personality disorder and being treated for recurrent depression and generalised anxiety disorder which didn’t fully remit in the 2 years she was open to the service.

She is followed through admission and investigations to a course of treatment with Bilateral ECT lasting for 12 sessions.

**Results:**

On Montgomery-Åsberg Depression Rating Scale the patient scored 34 before starting ECT treatment, and after completing the course scored 20. Clinical global impression rating rose from 6- severely ill, before course of ECT to 4- moderately ill, following.

Despite the potential multiple contributing diagnoses, the patient’s mental state and presentation improved in response to treatment with ECT.

**Conclusion:**

There is evidence of benefit in switching and augmenting antidepressants in older adults where there is treatment resistance (Lenze et al. 2023). This case supports the hypothesis that in treatment resistance in the elderly population we should persevere up to and including the use of ECT, until remission is achieved.

There’s a lack of research looking specifically at the impact of comorbid dependent personality disorder on efficacy of ECT for depression. Most of the existing evidence focusses on Emotionally unstable personality disorder.

This case study supports the hypothesis that there is evidence of treatment response with the use of ECT for treatment resistant depression regardless of comorbid dependent personality disorder.

